# Endobronchial Lipoma: A Rare Cause of Bronchial Stenosis or Obstruction

**DOI:** 10.1155/2023/2799436

**Published:** 2023-12-26

**Authors:** Jian Chen, Tao Xin, Lei Pan, Yan Li, Weisheng Qian, Jin Wei, Yan Yan, Yan Wang, Faguang Jin, Hua Jiang

**Affiliations:** ^1^Department of Respiratory and Critical Care Medicine, Tangdu Hospital, Fourth Military Medical University, Xi'an 710038, Shaanxi, China; ^2^Department of Respiratory and Critical Care Medicine, Shaanxi Provincial People's Hospital, Xi'an 710068, Shaanxi, China

## Abstract

Endobronchial lipoma (EL) is a rare benign tumor characterized by tracheobronchial smooth-surfaced mass, often resulting in bronchial obstruction without standard guidelines for management. This study seeks to clarify the clinical features and interventions of EL, aiming to improve its diagnosis and outcomes. A retrospective review was conducted on 28516 outpatients treated between January 2015 and December 2019 at the Department of Respiratory and Critical Care Medicine of the Second Affiliated Hospital of Air Force Medical University to collect patients diagnosed with EL. Their clinical, bronchoscopic, chest imaging, and histopathological features along with management were analyzed. Among the patients reviewed, nine were histopathologically diagnosed with EL, comprising seven males and two females. All EL patients exhibited noticeable symptoms, including cough (in eight patients), dyspnea (in six patients), fever (in three patients), expectoration (in two patients), chest pain (in two patients), hemoptysis (in one patient), and fatigue (in one patient). Chest CT abnormalities included endobronchial mass (in four patients), inflammatory exudation (in three patients), atelectasis (in three patients), and infiltration or consolidation (in two patients). In three patients, imaging showed fat density, directly leading to the diagnosis of EL. The EL lesions were distributed with six in the right lung and three in the left lung, all located within the first three subdivisions of the tracheobronchial tree. Treatment approaches varied, with one patient undergoing combined bronchoscopic resection and surgery. The remaining patients received bronchoscopic intervention such as electrosurgical snare resection, argon plasma coagulation (APC), cryotherapy, and holmium laser. Histopathological analysis confirmed the EL diagnosis. Finally, the mass removal restored bronchus patency. Taken together, EL symptoms lack specificity, necessitating reliance on histopathology for EL accurate diagnosis. Bronchoscopic interventions emerge as the preferred option for EL management, surpassing surgical approaches.

## 1. Introduction

Endobronchial lipoma (EL) is an exceedingly rare benign tumor, constituting approximately 0.1–0.5% of all lung tumors [1, 2]. The majority of EL cases originate from adipocytes in the submucosal layer of the main or lobular bronchi [2]. EL accounts for 3.2%–9.5% of the benign endobronchial tumors [3]. Despite their clinical and biological behaviour, these endobronchial masses can lead to tracheobronchial obstruction or stenosis, serving as the essential pathogenic factors that induce patients' symptoms and signs.

EL is more prevalent in middle-aged male, and significant risk factors include heavy smoking and obesity [4, 5]. Common clinical manifestations of EL include productive cough, hemoptysis, wheezing, obstruction, recurrent pneumonias, and bronchiectasis [5]. Diagnosis of EL is typically established through chest computed tomography (CT) and confirmed by histopathologic examination of bronchoscopic biopsy and/or surgically resected specimen [3, 4].

Tumor removal is the core treatment to restore airway function and improve the respiratory status. Increasing cases suggest that bronchoscopic intervention is a safe and effective option for EL [6–8]. The advantage of bronchoscopic resection lies in its less invasive feature, resulting in few complications compared with surgery [5]. As there are no standard guidelines for EL management, this study aims to provide a foundational strategy for the diagnosis and treatment of EL, by elucidating the clinical features and treatment outcomes of nine patients diagnosed with EL that caused bronchial stenosis or obstruction.

## 2. Materials and Methods

### 2.1. Study Design and Data Collection

This trial was conducted in accordance with the Declaration of Helsinki (as revised in 2013). Approval for this study was granted by the Ethics Committee of the Second Affiliated Hospital of Fourth Military Medical University (no. 2013027). All of the subjects signed the informed consent form before they underwent bronchoscopy. Given the retrospective nature of this study, informed consent for human research was not obtained from the participants. Waiver of the consent to use the patients' records was obtained from the Institutional Review Board, and this study was carried out after anonymization of the patients' data.

A retrospective review was conducted on 28516 outpatients treated between January 2015 and December 2019 from the Department of Respiratory and Critical Care Medicine of the Second Affiliated Hospital of Fourth Military Medical University. The objective was to identify patients diagnosed with EL, and a total of nine patients were included in this study. The average time required for EL diagnosis was 66.25 (ranging from 10 to 245) days. In the early stages, five cases (41.67%) were suspected of lung cancer, three cases (25%) were suspected of pneumonia, and only four cases were considered as EL. Medical records of these patients, including age, gender, complaints, smoking history, history of pulmonary diseases, sizes of the tumors, chest CT images, and bronchoscopic and histopathologic features, as well as managements and outcomes, were retrospectively analyzed. All patients were followed up for two years.

### 2.2. Statistical Analysis

All summary data are presented as the mean ± SD for quantitative variables. Categorical variables are described as number (percentage). Categorical variables (<5) were analyzed with Fisher's exact test, and continuous variables were assessed by Student's *t*-test. A difference of *P* < 0.05 was considered to be significant. All statistical analyses were conducted using SPSS 26.0.

## 3. Results and Discussion

### 3.1. Epidemiology

Among the 28516 patients, nine individuals were diagnosed with EL (overall incidence rate: 0.3%) in seven males and two females. As shown in Table 1, their age ranged from 42 to 66 years (the average age: 56.56 ± 9.04). Among the nine patients, comorbidities included chronic obstructive pulmonary disease (COPD) in one case, right lung tuberculosis in another, hypertrophic cardiomyopathy with atrial fibrillation in one, hypertension in another, and one patient had a history of pleural fiberboard stripping with chronic empyema. In addition, one case had been initially misinterpreted as asthma and another as lung cancer. Among the nine cases, six had a positive smoking history.

### 3.2. Clinical Presentation

As indicated in Table 1, the predominant symptom among the patients was chronic cough, observed in eight out of nine cases. Additional clinical complaints encompassed dyspnea (six out of nine), intermittent fever (three out of nine), sputum production (two out of nine), chest tightness and pain (two out of nine), hemoptysis (one out of nine), and fatigue (one out of nine). Patients were symptomatic for months to years. Abnormal findings on chest auscultation were noted in four patients, including absent respiration, wheezing, and stridor.

### 3.3. Diagnosis (Chest Imaging, Bronchoscopy, and Histopathology)

**Figure 1:**
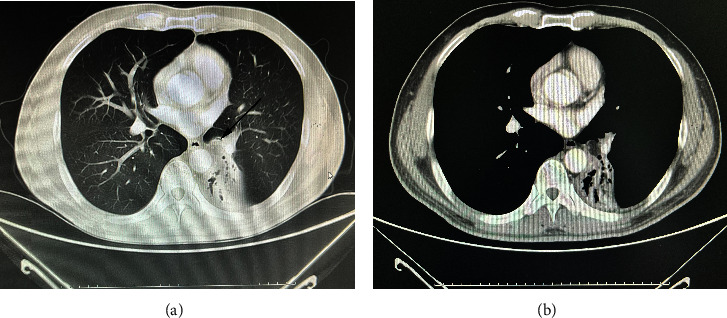
The findings of the endobronchial lipoma on chest CT showing a low attenuation lesion in the proximal bronchus of the left lower lobe on the lung (a) and mediastinal window (b).

All EL patients underwent chest CT scans at our hospital. As depicted in Figure 1 and Table 2, the CT images predominantly revealed the endotracheal and endobronchial homogenous soft tissue masses. These masses exhibited round or oval shapes, with well-defined boundaries against the surrounding mucosa. The densities of these masses were consistently low in the mediastinal window, distinctly contrasting with the surrounding normal airway tissue. Notably, they manifested characteristics of fat density, with CT values ranging from −110 to −120 Hounsfield units. Moreover, these masses were often accompanied by lobar or segmental atelectasis in eight out of nine cases, obstructive pneumonia in three out of nine cases, and bronchial stenosis in one out of nine cases. The CT images of one patient were atypical.

**Figure 2:**
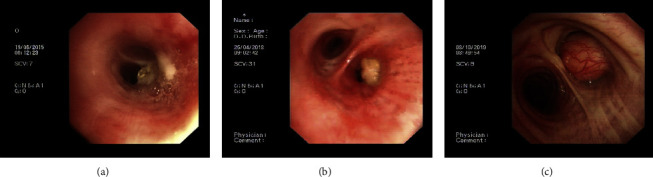
Bronchoscopic image showing the varying degrees of luminal stenosis due to endobronchial lipoma: airway mucosa bulge (a), bronchial stenosis (b), and bronchial obstruction (c).

Moreover, these masses were visible under bronchoscopy, appearing round and smooth surfaced with sizes ranging from 0.5 cm to 6 cm. As outlined in Table 3, their locations were primarily in the proximal bronchial tree, including two in the right main bronchus, two in the right upper bronchus, two in the right lower bronchus, two in the left upper bronchus, and one in the left lower bronchus. The majority of these masses were right sided (six out of nine). Generally, EL resulted in varying degrees of luminal stenosis, including airway mucosa bulge (one out of nine), bronchial stenosis (two out nine), and bronchial obstruction (six out of nine) (Figure 2).

**Figure 3:**
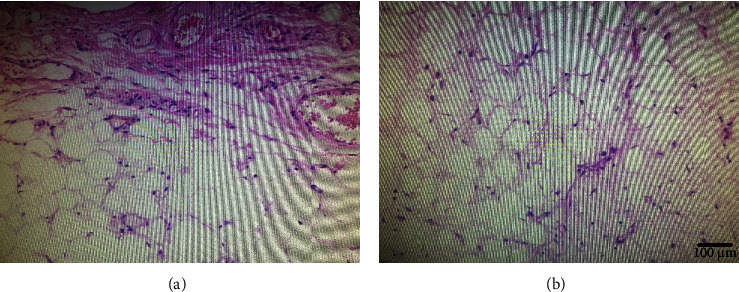
The representative H&E images showing the fibrous connective tissue and adipose tissue (a). The adipose tissue showing numerous adipocytes with marked variation in size (b).

Histopathological examination of these masses, as shown in Figure 3, revealed polypoid tumors consisting of mature adipocytes covered with the pseudostratified ciliated columnar epithelium. Hematoxylin and eosin (HE) staining further highlighted the respiratory epithelium overlying fibrous connective tissue and fat. On the mass surface, fibrous tissue resembled inflammatory granulation tissue, showing increased vascularity and infiltration of mixed acute and chronic inflammatory cell. The underlying adipose tissue showed adipocytes with marked variation in size.

### 3.4. Management and Complications

**Figure 4:**
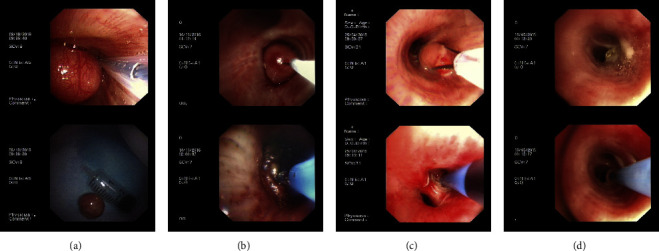
Multiple endoscopic interventions were performed to remove the endobronchial lipoma. Electric snare resection (a), electric snare resection combined with APC (b), electric snare resection combined with cryotherapy (c), and APC combined with cryotherapy (d).

Flexible bronchoscopic intervention alone was performed in eight cases. Nevertheless, bronchoscopic intervention combined with surgery was carried out in one case. In the latter instance, a right lower lobe tumor was detected by chest CT. A rigid bronchoscope combined with a flexible one was utilized under general anesthesia. A yellow spherical mass in the left lower lobe was identified. Electric snare resection was employed to remove this mass, and argon plasma coagulation (APC) was used to cauterize the base. One month later, chest CT revealed dorsal segment atelectasis of the left lower lobe, leading to left lower lobectomy through thoracic surgery. Postoperative histopathologic examination confirmed the presence of lipoma with hamartoma. As shown in Table 3 and Figure 4, these interventions were carried out under general anesthesia (six out of nine) or local anesthesia (three out of nine). The endoscopic interventions included APC in all cases, electric snare resection in six out of nine, cryotherapy in five out of nine, and holmium laser in two out of nine. Minor bleeding occurred in two cases (22.22%), and hemocoagulase was administered under a bronchoscope to stop bleeding during the operation. For two cases (22.22%) with moderate bleeding, hemocoagulase was perfused through a bronchoscope and the intravenous pituitrin was also used. There were no other complications.

### 3.5. Outcome and Prognosis

As shown in Figure 4 and Table 4, the abnormal stenosis of the bronchial lumen had been improved, with either nonstenosis or a reduction to ≤25% following interventions. Moreover, the shortness of breath of 7 patients (78%) had completely mitigated, with only two patients (22%) experiencing this symptom during brisk walking. A thorough follow-up spanning two years was conducted, with a primary focus on their symptoms. If the shortness of breath score was equal to or greater than grade II, accompanied by any other symptoms, bronchoscopy would be performed on the third month. Otherwise, patients would undergo chest CT scans at the end of the follow-up period to assess airway stenosis. After two years of follow-up, the bronchial lumen in all patients remained unobstructed, with six patients exhibiting nonstenosis and three patients showing ≤25% stenosis.

## 4. Discussion

Endobronchial lipoma (EL) is a rare benign lung tumor, typically occurring in middle-aged and elderly men. Its clinical symptoms are nonspecific, often leading to misdiagnosis [9]. Obesity and smoking are recognized as major risk factors [10, 11]. EL is characterized by expansive growth, potentially causing partial or complete obstruction of pulmonary segments, lobes, or even the main bronchi. In this study, six cases were located in the right lung and three cases in the left lung, consistent with previous literature studies, indicating a higher prevalence in the right lung [2, 12]. EL is typically solitary and unilateral, although multiple cases have been reported [7]. In our study, all the nine cases were all solitary. EL could be divided into the proximal and distal types [13], with the former occurring in the central airway, including the trachea, carina, left and right main bronchi, and middle segment of the right lung. The latter type occurs in the lobar and segmental and subsegmental bronchi. Clinical symptoms, such as cough, shortness of breath, and fever from obstructive pneumonia, are related to the EL size, location, and degree of bronchial obstruction [11]. The average time from the onset of symptoms to diagnosis was 66.25 days, possibly reflecting a lack of attention to symptoms by the patients themselves.

The primary imaging manifestation of EL is a soft tissue mass in the trachea or bronchi, which could cause atelectasis by blocking the bronchial lumen. The diagnosis of EL could be indicated when the masses present fat density on chest CT [6]. In this study, three cases (33.33%) showed homogenous fat density and were diagnosed as bronchial lipoma. The CT value of tracheobronchial lipoma typically falls between −40 and −120 HU [7]. Chest CT proves to be more valuable than bronchoscopy in displaying the presence or absence of surrounding bronchial wall invasion [6]. Nevertheless, it remains a challenge to distinguish endobronchial lipoma from hamartoma by CT as both are predominantly composed of adipose tissues [7]. Given that adipose tissues are often present outside the cartilage ring of large bronchi, it is noteworthy that the vast majority of endobronchial lipomas tend to occur in the central airway [14]. The distinctive low density of lipomas, in contrast to other tumors [15] or inflammatory conditions, is helpful for an initial differential diagnosis. The locations and sizes of these masses could determine whether the airway patency has been compromised. The integration of chest CT findings with the patient's symptoms proves to be a helpful approach in ruling out other potential airway diseases, such as bronchial cancer or inflammatory conditions.

Bronchoscopy plays a crucial role in identifying EL. In this study, the chest CT of one case (11.11%) failed to detect an endobronchial mass that was identified by the bronchoscopy. This suggests that bronchoscopy and chest CT cannot be replaced by each other. The feature of EL under bronchoscopy is a spherical mass with observable neovascularization on the surface. EL presents as a hard, brittle, and fragile lesion. When the lesion was located at or below the segmental bronchus, the bronchoscopic exploration would be limited. Generally, the masses are separated from the luminal mucosa, causing bronchial stenosis or obstruction [16]. In addition, EL mainly originates from the submucosal adipose tissue, which seems much smoother and is covered by the normal bronchial mucosal columnar epithelium or the inflammatory squamous epithelium. These prevent the EL being clamped and diagnosed accurately [17]. In this study, all patients were diagnosed by histopathologic examination, which made up for the low positive rate of biopsy.

The advancement of interventional pulmonology has positioned bronchoscopic intervention as a crucial choice for EL treatment. This intervention can be conducted using both rigid and flexible bronchoscopes, employing specific devices such as high-frequency electrosurgical resection, argon plasma coagulation (APC), cryotherapy, and laser [6]. In our study, high-frequency electrical snare ligation emerged as the most frequently used method. This technique involves a hybrid mode of electrocoagulation and electrocautery, effectively preventing bleeding during resection. Careful examination of the lesion base is crucial, aiming to clear it as far as possible without delving too deeply. Anesthesia methods depend on tumor characteristic, with general anesthesia recommended for large tumors with rich vascularity. The combination of rigid and flexible bronchoscopes is employed due to its effective ventilation and large working channel [18]. Surgery remains an important option for removing endobronchial lipoma. In our study, only one lesion could not be completely removed through high-frequency electrical snare ligation, leading to surgery resection. A previous article compiling 64 EL cases (33 cases previously reported in 30 different articles and 31 case reports presented at thoracic meetings in Japan) revealed a different treatment landscape [2]. In that study, forty patients underwent surgical resection, while 17 cases received bronchoscopic intervention. Moreover, the bronchoscopic interventions included Nd-YAG laser and electric snare resection in that study, which were also distinct from those in our study. These differences in therapeutic concepts may reflect the evolving landscape of interventional devices in the field.

## 5. Conclusions

EL is a relatively rare benign tumor with nonspecific clinical manifestations. Its definitive diagnosis mainly relies on histopathology. Bronchoscopic intervention stands out as the preferred choice, proving effective in alleviating obstructive symptoms with low risks and fewer complications. However, in cases where EL is concomitant with a malignant tumor, bronchoscopic intervention may not completely eradicate the lesions, making surgery a crucial alternative.

## Figures and Tables

**Table 1:** The essential characteristics of the nine EL patients.

IDs	Gender	Age (years)	Comorbidities	Misdiagnosis	Smoking history (Y/N)	Clinical presentations
13899282	Female	45	N/A	Asthma	N	Chronic cough, dyspnea, chest tightness, and pain
14599853	Male	45	N/A	N/A	Y	Chronic cough and intermittent fever
11495609	Male	66	N/A	Lung cancer	Y	Dyspnea, intermittent fever, chest tightness and pain, and fatigue
14159812	Female	63	Hypertrophic cardiomyopathy	N/A	N	Chronic cough sputum and hemoptysis
14358735	Male	66	Right pulmonary tuberculosis	N/A	Y	Chronic cough and sputum
14456406	Male	55	N/A	N/A	Y	Chronic cough and dyspnea
14924336	Male	61	Hypertension	N/A	Y	Chronic cough and dyspnea
60224720	Male	66	COPD	N/A	N	Chronic cough and dyspnea
13321732	Male	42	Chronic empyema	N/A	Y	Chronic cough, dyspnea, and intermittent fever

**Table 2:** Chest CT findings of the nine patients with endobronchial lipoma.

IDs	Endobronchial masses	Position	Morphology	Lesion density (HU)	Size (cm)	Accompanying changes
13899282	Y	Right upper bronchus	Round	−110	2.6	Atelectasis
14599853	Y	Left lower bronchus	Round	−115	3.2	Obstructive pneumonia
11495609	N/A	N/A	N/A	N/A	N/A	Atelectasis and obstructive pneumonia
14159812	Y	Right upper bronchus	Oval	−110	1.8	Bronchial stenosis
14358735	Y	Right main bronchus	Round	−120	3.9	Bronchial stenosis
14456406	Y	Left upper bronchus	Round	−118	3.5	Atelectasis
14924336	Y	Right lower bronchus	Round	−115	2.9	Atelectasis
60224720	Y	Right main bronchus	Round	−110	4.2	Atelectasis
13321732	Y	Right lower bronchus	Round	−116	3.2	Atelectasis and obstructive pneumonia

**Table 3:** Bronchoscopic findings and interventions of endobronchial lipoma.

IDs	Site	Side	Luminal stenosis	Anesthesia	Management	Complications
13899282	Upper bronchus	Right	Bronchial obstruction	Local	Electric snare resection + cryotherapy + APC	Minor bleeding
14599853	Lower bronchus	Left	Bronchial obstruction	General	Electric snare resection + APC + surgery	N/A
11495609	Upper bronchus	Left	Bronchial obstruction	General	Cryotherapy + APC	Moderate bleeding
14159812	Upper bronchus	Right	Bronchial obstruction	General	Holmium laser + APC	N/A
14358735	Main bronchus	Right	Bronchial stenosis	Local	Electric snare resection + cryotherapy + APC	Moderate bleeding
14456406	Upper bronchus	Left	Mucosa bulge	Local	Electric snare resection + cryotherapy + APC	N/A
14924336	Lower bronchus	Right	Bronchial obstruction	General	Electric snare resection + APC	N/A
60224720	Main bronchus	Right	Bronchial stenosis	General	Electric snare resection + cryotherapy + APC	Minor bleeding
13321732	Lower bronchus	Right	Bronchial obstruction	General	Holmium laser + APC	N/A

**Table 4:** Outcome and prognosis of the nine patients with endobronchial lipoma.

IDs	Bronchial stenosis		Shortness of breath		Prognosis
	Before	After	Before	After	
13899282	V	0	2	0	I
14599853	V	I	3	0	I
11495609	V	I	1	0	I
14159812	IV	I	3	1	II
14358735	V	0	4	1	I
14456406	V	0	3	0	I
14924336	V	0	2	0	I
60224720	V	0	4	0	I
13321732	III	0	1	0	II

ATS CT grading of bronchial stenosis: 0, nonstenosis; I, ≤25%; II, >25% and ≤50%; III, >50% and ≤75%; IV, >75% and ≤90%; and V, >90% and ≤100%. The index of shortness of breath: 0, normal; 1, shortness of breath during brisk walking; 2, shortness of breath during walking at normal speed; 3, stopping walking due to shortness of breath when walking at normal speed; and 4, shortness of breath after slight activity; Prognostic grading: I, bronchial stenosis remission ≥75% for 3 months; II, bronchial stenosis remission >50% and ≤75% for 3 months; III, bronchial stenosis remission >25% and ≤50% for 3 months; and IV, bronchial stenosis remission ≤25%, mucosal lesions are still present.

## Data Availability

The data used to support the findings of this study are available from the corresponding author upon reasonable request.
